# A time and equipment saving tip for difficult urethral catheterizations

**DOI:** 10.1308/rcsann.2014.96.1.79

**Published:** 2014-01

**Authors:** A Gupta, P Hughes, C Coker

**Affiliations:** Brighton and Sussex University Hospitals NHS Trust,UK

Inability to a pass a urethral catheter is a common scenario in urology. Options include inserting a suprapubic catheter, using an introducer or railroading a catheter over a guidewire inserted via flexible cystoscopy. We describe a novel bedside technique using the atraumatic hydrophilic properties of Terumo (Egham, UK) guidewires. Insert the wire blindly down the urethra until two-thirds has been passed, guaranteeing that it lies in the bladder. Perforate the tip of a Foley catheter with a 16G intravenous cannula and railroad it over the guidewire. This safe technique negates the need for more invasive methods in

